# Strategies to Characterize Fungal Lipases for Applications in Medicine and Dairy Industry

**DOI:** 10.1155/2013/154549

**Published:** 2013-06-24

**Authors:** Subash C. B. Gopinath, Periasamy Anbu, Thangavel Lakshmipriya, Azariah Hilda

**Affiliations:** ^1^Center for Advanced Studies in Botany, University of Madras, Guindy Campus, Chennai, Tamil Nadu 600025, India; ^2^Electronics and Photonics Research Institute, National Institute of Advanced Industrial Science and Technology, Central 5, 1-1-1 Higashi, Tsukuba, Ibaraki 305-8565, Japan; ^3^Department of Biological Engineering, College of Engineering, Inha University, Incheon 402-751, Republic of Korea; ^4^Department of Mathematics, SBK College, Madurai Kamaraj University, Aruppukottai, Tamil Nadu 626101, India

## Abstract

Lipases are water-soluble enzymes that act on insoluble substrates and catalyze the hydrolysis of long-chain triglycerides. Lipases play a vital role in the food, detergent, chemical, and pharmaceutical industries. In the past, fungal lipases gained significant attention in the industries due to their substrate specificity and stability under varied chemical and physical conditions. Fungal enzymes are extracellular in nature, and they can be extracted easily, which significantly reduces the cost and makes this source preferable over bacteria. Soil contaminated with spillage from the products of oil and dairy harbors fungal species, which have the potential to secrete lipases to degrade fats and oils. Herein, the strategies involved in the characterization of fungal lipases, capable of degrading fatty substances, are narrated with a focus on further applications.

## 1. Introduction

**Figure 1:**
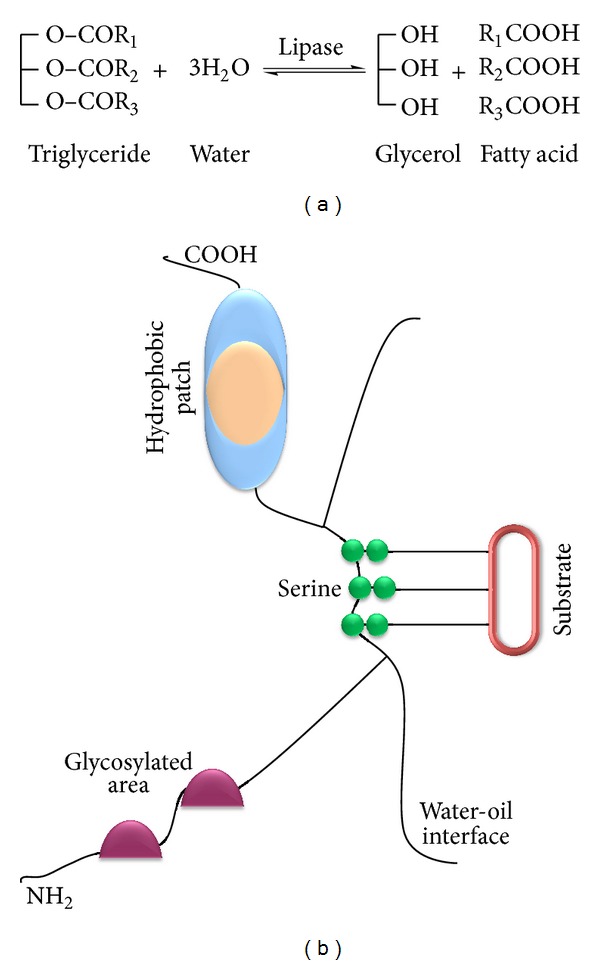
(a) Hydrolysis of triglyceride by lipase [1]. Upon hydrolysis triglyceride converts into glycerol and fatty acid. (b) Representation of a molecule of lipase with its features. The substrate can be any triglyceride [2]. Substrate interactive regions are displayed.

Lipases are defined as triacylglycerol acyl hydrolases (EC 3.1.1.3) and are involved in the hydrolysis of fats and oils to yield glycerol and free fatty acids [1] (Figure 1(a)). Lipases belong to the class of serine hydrolases and do not require any cofactor. Under natural conditions, lipases catalyze the hydrolysis of ester bonds at the interface between an insoluble substrate phase and the aqueous phase where the enzyme remains dissolved [2] (Figure 1(b)). Lipases are involved in conversion reactions, such as esterification, interesterification, transesterification, alcoholysis, acidolysis, and aminolysis [3]. Many microorganisms such as bacteria, yeasts, molds, and a few protozoa are known to secrete lipases for the digestion of lipid materials [1, 4–12]. Microbes, being ubiquitous in distribution, are highly successful at surviving in a wide range of environmental conditions owing to their great plasticity and physiological versatility and have been the subject of several reviews [13, 14]. Due to efficient enzyme systems, microbes thrive well in inhospitable habitats [15]. With mechanisms for adapting to environmental extremes and for the utilization of their trophic niche, the ability of microorganisms to produce extracellular enzymes is of great survival value [16]. Among different microbial enzymes, lipases are widely documented among bacteria, fungi, plants, and animals [17, 18].

Extracellular secretion has been well studied for a number of fungi, primarily zygomycetes [19], hyphomycetes [20], and yeasts [21, 22]. Lipase production has also been reported for some ascomycetes [23] and coelomycetes [24]. Lipolytic activity has been observed in *Mucor *spp. [25, 26], *Lipomyces starkeyi* [27], *Rhizopus *spp. [26, 28–30], *Geotrichum candidum* [25, 31–34], *Pencillium *spp. [9, 28, 35, 36], *Acremonium strictum* [37], *Candida rugosa* [38], *Humicola lanuginosa* [39], *Cunninghamella verticillata* [40], and *Aspergillus *spp. [11, 41]. Considering the importance of fungal lipases, their applications are discussed and the techniques involved in lipase generation have been gleaned recently [1].

Fungi are involved in the degradation of undesirable materials or compounds converting them into harmless, tolerable, or useful products. The undesirable materials include sewage waste from domestic and industrial complexes and plant, animal, and agricultural wastes, oil spills, and dairy waste. The role of fungi in bioremediation processes in varied environments has been well documented [16, 30, 42, 43]. There has been an increasing awareness of potentially harmful effects of the worldwide spillage of oil and fatty substances in both saline and fresh waters. Domestic waste is also considered as the pollutants as it has a high amount of fatty and oil substances. Industrial and domestic wastes harbor fungal species of greater potential in degrading fats and oils. Besides waste disposal, bioconversion by fungal activity results in the production of a vast number of useful substances. Thus, waste can be converted into a resource. Bearing in mind the importance of lipolytic fungal enzymes from different disposal sources, this overview focuses on strategies to characterize the fungal lipases with an emphasis on a wide range of applications.

## 2. Lipolytic Fungal Species from Oil-Spill Wastes

**Figure 2:**
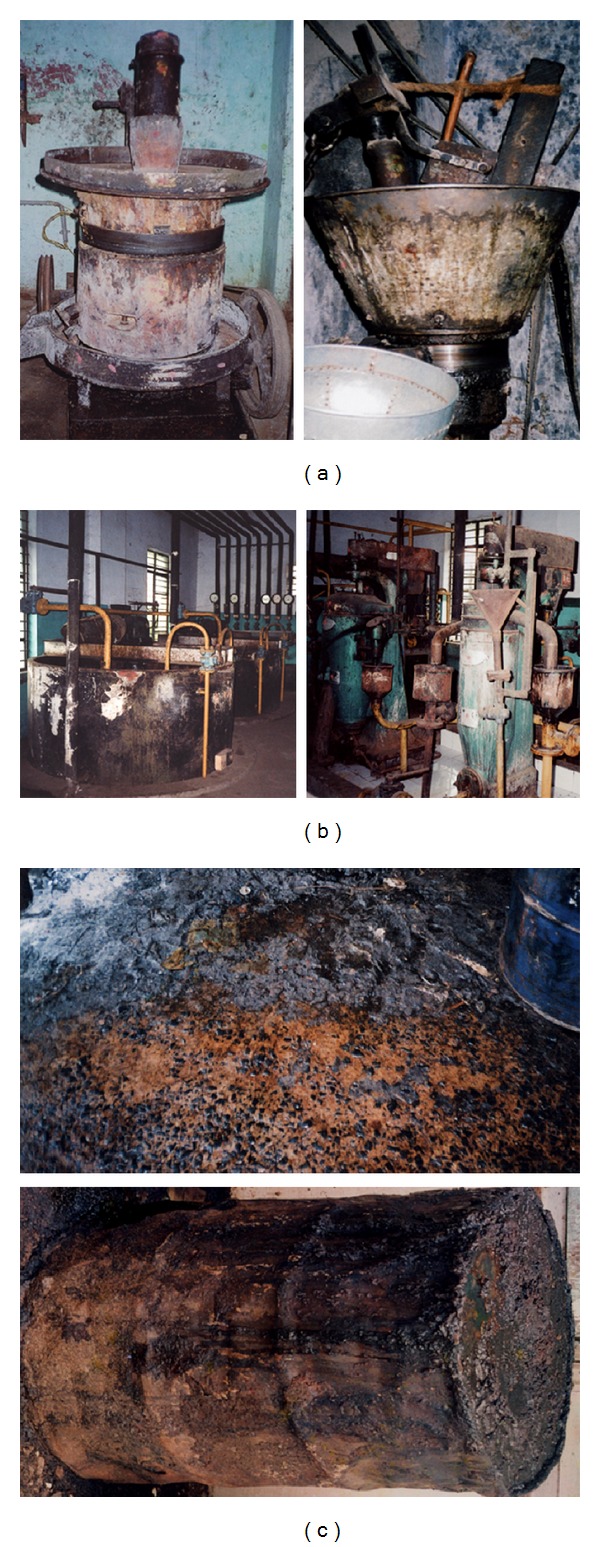
Oil production and spillage. (a) Crushing system in small-scale oil production. (b) Crushing system in large-scale oil production. (c) Spillage from oil production points. These releasing points are potential sources of environmental issues.

Due to usage of vegetable oils for cooking, these oils are released into the open environment both at the production level and by domestic users. To keep the environment clean, these oils should be degraded by using environmentally-friendly technology. Several oil-industries have been established at both small scale (Figure 2(a)) and large scale (Figure 2(b)). Oil spillages from these production points (Figure 2(c)) cause a hindrance in ensuring environmental hygiene due to the formation of clogs in drain pipes [44]. Cleanup and recovery of oil wastes is difficult and depends upon many factors, including the type of oil spilled, the temperature of water affecting evaporation, and biodegradation. Microbial degradation is one of the most important events to ameliorate oil pollution in the environment. Fungi that produce lipases are found in diverse habitats including oil-contaminated soils, wastes around oil processing factories, domestic waste points, and dairy products [27]. Gopinath et al. [16] have isolated 34 fungal species from oil-spill contaminated soils, collected in major cities of India. These species were tested for their survival with the changes in seasons. Twelve fungal species from oil-mill effluent composts at Nsukka have been studied and it was found that *Aspergillus *spp. are more common; however, the higher lipase producers are *Trichoderma* sp. followed by *Aspergillus* spp. [9]. D'Annibale et al. [45] used olive mill waste water as the substrate to determine lipase production. Lipase producing fungal species were also recovered from compost heaps, coal tips, and industrial wastes [43]. Cihangir and Sarikaya [42] have isolated *Aspergillus *sp. from the soil samples collected in Turkey. Extracellular lipase of *Rhizopus *sp. isolated from oil-contaminated soil was recently characterized [30].

## 3. Screening Lipase Production on Agar Solid Surface

Studies on mycoflora are significant as they could harbor species of the highest potential for degradation. The industrial demand for new lipase sources continues to stimulate the isolation and screening of new lipolytic microorganisms. In view of the interesting applications of microbial lipases, it could be of tremendous value to screen and identify microorganisms of highest potential for the biodegradation of oils and fats. Although, different screening strategies have been proposed for the determination of lipase activity, assays using agar plates are highly recommended, because it is an easier method with lower cost. Assay using agar plates are performed due to the fact that activities for lipases are hard to determine because of the water-soluble enzyme acting on substrates which are insoluble [46, 47].

**Figure 3:**
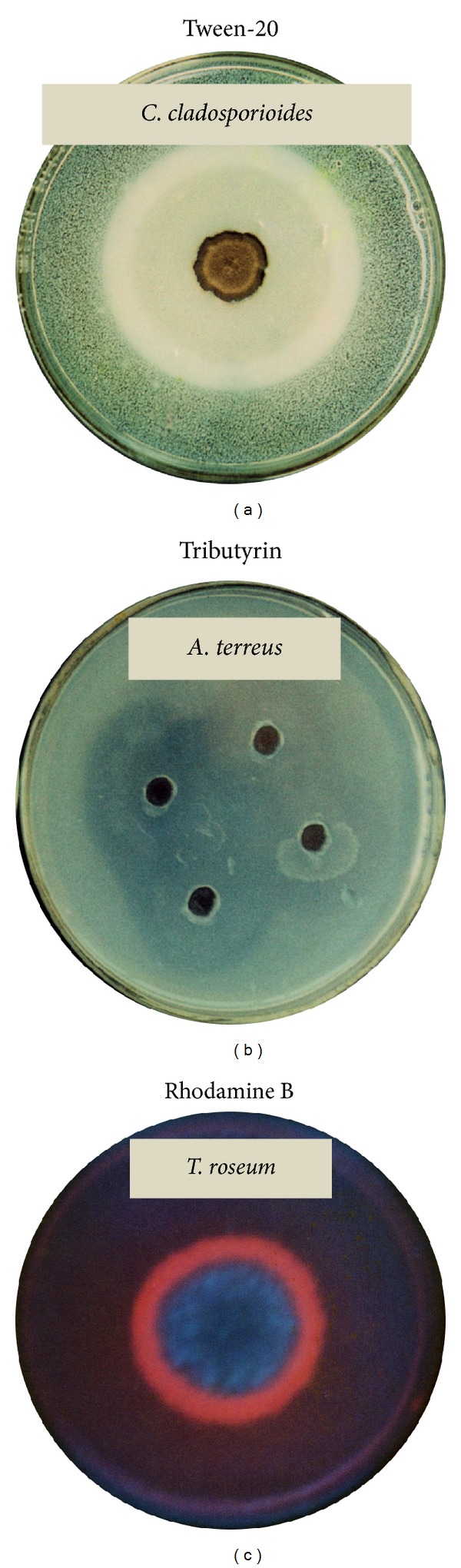
Agar plate screening for lipases. Using the substrates (a) Tween, (b) tributyrin, and (c) vegetable oil. In the Tween method, formation of calcium crystals was observed. The tributyrin method shows a clear zone, whereas in the Rhodamine method, formation of fluorescence with fatty substrate was observed under UV illumination. Active zones are increasing with a period of incubation time and these zones can be measured.

To isolate fungal species from the oil-spill contaminated soils, screening studies were performed by Gopinath et al. [16, 40] using different substrates on agar plates. These methods with different substrates include Tween-20 (Figure 3(a)), tributyrin (Figure 3(b)), and vegetable oil in the presence of Rhodamine (Figure 3(c)). Due to the oil rich environments of the substrates, special attention was given to screening of lipolytic enzymes. On the Tween-20 substrate, a visible precipitate appeared due to the deposition of calcium salt crystals formed by the liberated fatty acid by the action of lipase or by clearing of such a precipitate due to complete degradation of the fatty acid salts. Brockman [48] suggested that the primary role of calcium ions is to remove fatty acids formed during hydrolysis as insoluble calcium soaps, and thus changing the substrate-water interface relationship to favorable conditions for enzyme action. The development of a clear crystal zone of Tween-20 around the fungus was also an indication of lipolytic activity, and this zone can be measured. Using Tween-20 as the substrate, Salihu et al. [36] screened different fungal species for the production of lipases. Another substrate, tributyrin, is convenient because it is easily dispersed in water by shaking or stirring without the addition of any emulsifiers. Tributyrin is a very strong surface-active substance, and its hydrolysis can be followed by measuring the increase in the diameter of the clear zone. Nevertheless, the observed zones of clearing could be the activity response of nonspecific esterases, which may have little or no activity against the long-chain triglycerides [49]. Hence, it is imperative to use another method to confirm true lipase activity. Tributyrin agar plates were used to investigate lipase production by new strains and 18 strains were found to be positive [42]. Using tributyrin formation of the clear zone around the fungal colony showed different mutant strains that produced extracellular lipases [50]. By using Tween-20 and tributyrin substrates, lipolytic activity (high and moderate activity) was evidenced by 19 and 32 species, respectively [51]. The lipolytic potential of this fungus was also confirmed by the Rhodamine method because the enzyme will fluoresce with orange compound (Figure 3(c)) as reported by Kouker and Jaeger [52]. Furthermore, Hou and Johnston [53] as well as Lee and Rhee [54] proved that this method is highly sensitive and reliable as a lipase assay. In a recent study, the Rhodamine method with olive mill wastewater was used to determine the production of lipases by *Aspergillus ibericus* [11]. Savitha et al. [3] used Rhodamine fluorescence-based assay to screen 32 fungal species from different sources. Our previous results provide very useful information about the degradation of vegetable oils by *Cunninghamella verticillata* in the presence of Rhodamine [51]. Based on the above screening strategies, Gopinath et al. [16] revealed the following fungi as potential candidates that secrete enzymes lipases, *Absidia corymbifera, Aspergillus fumigatus*, *Aspergillus japonicus*, *Aspergillus nidulans, Aspergillus terreus, Cunninghamella verticillata, Curvularia pallescens, Fusarium oxysporum, Geotrichum candidum*, *Mucor racemosus*, *Penicillium citrinum*, *Penicillium frequentans*, *Rhizopus stolonifer*, and *Trichoderma viride*. They also conducted screening studies for other enzymes and confirmed that some of the isolated fungal species could also secrete amylases, proteases, and cellulases in addition to lipases, representing the ability of these species to survive in a wide range of environmental substrates [16]. These observations provided interesting perspectives, demonstrating that fungi isolated from oil-rich environments represent a source of several enzymes potentially exploitable for biotechnological purposes. In another study, 59 fungi were screened by measuring the formation of halos on the agar plate used for lipase screening [55]. Preliminary screening studies for lipase production by fungi were also carried out on agar plates using olive oil or emulsified tributyrin by gum arabic [56]. Kumar et al. [57] screened fungi with bromophenol blue dye supplemented agar plates with olive oil as the substrate.

## 4. Degradation of Oils by Lipases

The use of specific microbial lipases to catalyze interesterification reactions became considerable interest because of its advantages over chemical catalysts. Traditionally, fatty acids are manufactured by the hydrolysis of oils at high temperature and pressure. However, lipase hydrolysis is an energy saving process because oil degradation of fatty acids (the reaction) can be carried out at room temperature and pressure [58]. Industrially important chemicals manufactured from fats and oils by chemical processes could be produced by lipases with rapidity and better specificity under mild conditions [59, 60]. Lipases are one among several kinds of extracellular enzymes that perform the function of recycling large quantities of insoluble organic material in nature [61]. Apart from numerous applications such as transesterification [62, 63], ester synthesis, production of biosurfactants [64], and application in food and dairy industry [35, 65, 66], the enzyme lipase has a proven role as a useful interesterification catalyst. Interesterification is a technology by which fatty acids within a triacylglycerol molecule can be interchanged with regard to their positional distribution. The process of fat splitting, along with interesterification, is an essential tool in the manufacture of new tailor made fats and oils. Enzyme-catalyzed reactions of lipids are of considerable interest in view of their possible applications in the biotechnology of fats and oils. The technique of fat splitting plays an important role in the manufacture of soaps and other industrial products like candles from conventional minor oils. Different isolates from oil mill effluent have been tested for their ability to degrade the different oils and the potential of individual species varied with the type of fatty acid residues in the oil (Table 1). From this study, it was revealed that the behavior of lipases from different fungal species is different in terms of their biochemical characteristics. Teng and Xu [67] analyzed the production of lipase from *Rhizopus chinensis* under experimental conditions and Bapiraju et al. [68] performed a similar study with mutants of *Rhizopus* sp. Studies have also been documented with lipase from *Penicillium* spp. [69, 70]. Extensive review on the production of lipases from different microbes has been published [71].

## 5. Purification Strategies for Lipases

**Figure 4:**
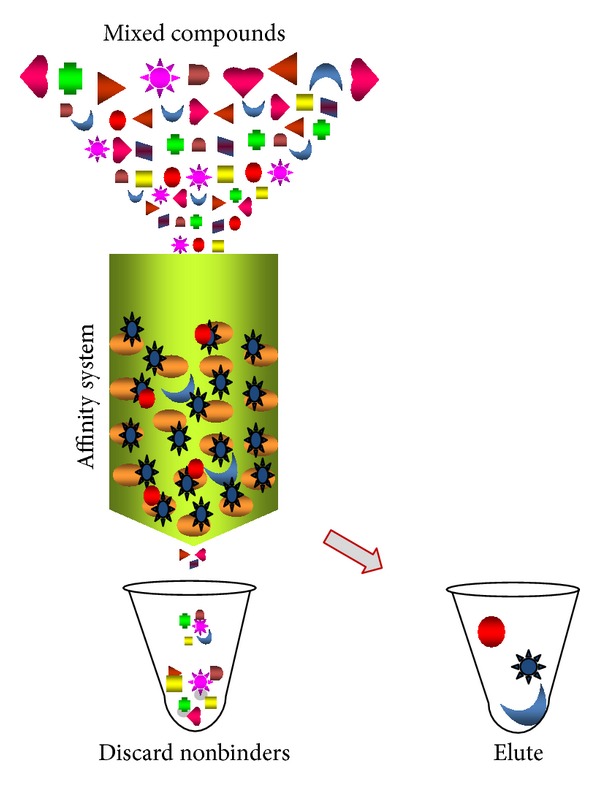
Purification of lipase using an affinity system. Separation of lipase from mixed compounds is indicated. Bound lipase can be eluted by creating stringent conditions.

Knowledge of the purified lipase activities to be used for biotechnological purposes is mandatory, and it can be the basis or other applications. Various purification strategies have been reviewed for the lipase enzyme [72–74]. In the case of extracellular lipases, it is primarily important to remove other contaminants from the compound mixture containing lipase by suitable strategy (Figure 4). The conventional purification strategies give a low yield due to a large hydrophobic surface near the active site. Novel purification steps are mandatory to increase overall enzyme yields and it could be achieved by opting an appropriate chromatography system. One of the choices is hydrophobic interaction chromatography and it is considered as a common strategy [75–77]. In addition to this, ion exchange and gel filtration chromatography are commonly preferred methods [75, 78–80]. A reversed micellar system, membrane processes, immunopurification, hydrophobic interaction chromatography with an epoxy-activated spacer arm (ligand), column chromatography using polyethylene glycol (PEG)/sepharose gel, and aqueous two-phase systems are also recommended [81]. Kumarevel et al. [82] reported a stepwise purification strategy for fungal lipases to remove other components released from the fungus *Cunninghamella verticillata* extracellularly, using acetone precipitation as the important step. To avoid many steps in this study and to minimize the impurities as much as possible the experiment was repeated with 50% acetone saturation with a gradual increments of 5% acetone. Using the above methods, many lipases from different microorganisms have been reported, and molecular masses of 31 and 19 kDa have been reported for the lipase of *Aspergillus niger* by Hofelmann et al. [83], 21.4 kDa for *Rhodotorula pilimena* [84], 30 kDa for *Rhizopus japonicus* [85], 51 kDa for *Pichia burtonii* [86], 25 kDa for *Aspergillus oryzae* [87], 49 kDa for *Mucor hiemalis* [88], 35.5 kDa for *Aspergillus niger* [89], 49 kDa for *Cunninghamella verticillata* [40], and 32 kDa for *Geotrichum candidum* [34]. Different strategies for lipase purification with the varied sources were recently described in detail by Singh and Mukhopadhyay [1], and it seems that the production of lipases from fungal species results in different molecular sizes, due to variations in the number of amino acid residues. Saxena et al. [73] summarized the purification strategies for microbial lipases. Overall, traditional purification strategies are considered time consuming with lower yields and the trends are moving towards aqueous two-phase extraction, and purification in ionic liquids and purification based on lipase-lipase interaction [10]. 

## 6. Statistical Calculations

**Figure 5:**
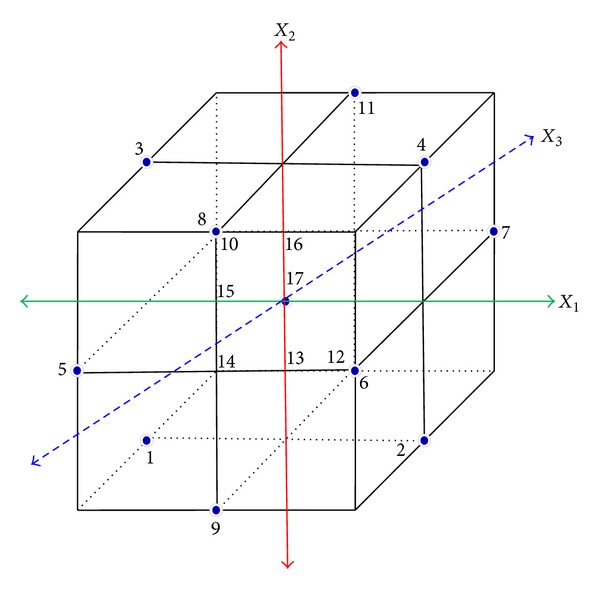
Box-Behnken design for experiments. Different combinations at the midpoints used for experiments are shown with 3 experimental parameters and 17 experiments run. A number of experiments vary with the number of experimental parameters.

Statistical calculations were focused in the past, due to their reliable prediction for the experimental conditions for enzyme studies to be optimized [34, 40, 90–92]. In statistics, response surface methodology (RSM) has referred the relationships between several explanatory variables and one or more response variables. This method was introduced initially by Box and Wilson [93]. The main idea of RSM is to get an optimal response by using a sequence of designing experiments, and it was suggested to use a second-degree polynomial model to perform RSM. Box-Behnken design experiments are one of the most common, and this is an independent quadratic design without an embedded factorial design. Different combinations of midpoints are used for experiments; for example, with 3 experimental parameters, 17 experiments can be run and it yields a predicted result (Figure 5). The Box-Behnken design is where the outcome unit (*Y*) is related to experimental variables by a response equation,

(1) $$\begin{array}{c} Y\;=\;f\left(X_{1},X_{2},X_{3},\ldots ,X_{k}\right). \end{array}$$

As mentioned above a second-degree quadratic polynomial is used to represent the function in the range of interest,

(2) $$\begin{array}{c} Y=R_{0}+\sum_{i=1}^{k}R_{i}X_{i}+\sum_{i=1}^{k}R_{ii}X_{i}^{2}+\sum_{i=1,i<j\;}^{k-1}\sum_{j=2}^{k}R_{ii}X_{i}X_{j}+\varepsilon , \end{array}$$

where  *X*_1_, *X*_2_, *X*_3_,…, *X*_*k*_ are the input variables which effect the response *Y*, *R*_0_, *R*_*i*_, *R*_*ii*_, and *R*_*ij*_ (*i* = 1 − *k*,   *j* = 1 − *k*) are the known parameters, and **ε** is the random error. A second-order model is designed such that the variance of *Y* is constant for all points equidistant from the center of the design (Figure 5). The validity of the model can be determined based on Student's *t*-test. The Fisher-test, *P* value, *t*-test, and *R* ^2^, and so forth can be used to evaluate the model as well as to determine the optimal processing conditions. The Fisher-test with a very low probability value (*P*_model_ > *F* = 0.0001) showed that the regression model had a very high significance. The model reliability of fit was checked by means of the determination coefficient (*R* ^2^). This model fits the experimental range studied perfectly when the value of  *R* ^2^ is adjusted to nearly one. Using the Box-Behnken design, conditions were optimized for lipase production by *Geotrichum candidum* [34] and optimization of purified lipase from *Cunninghamella verticillata* for physical parameters was also shown [40]. Using these optimized conditions, the purification steps were reduced and purified lipase was used for crystallization studies [82]. Other than Box-Behnken, the Plackett-Burman design and the Luedeking-Piret model can also be used for different optimization studies. Statistical design experiments by Plackett-Burman were used to evaluate the production of lipase by* Candida rugosa *[1].

## 7. Biosensors for Lipase

**Figure 6:**
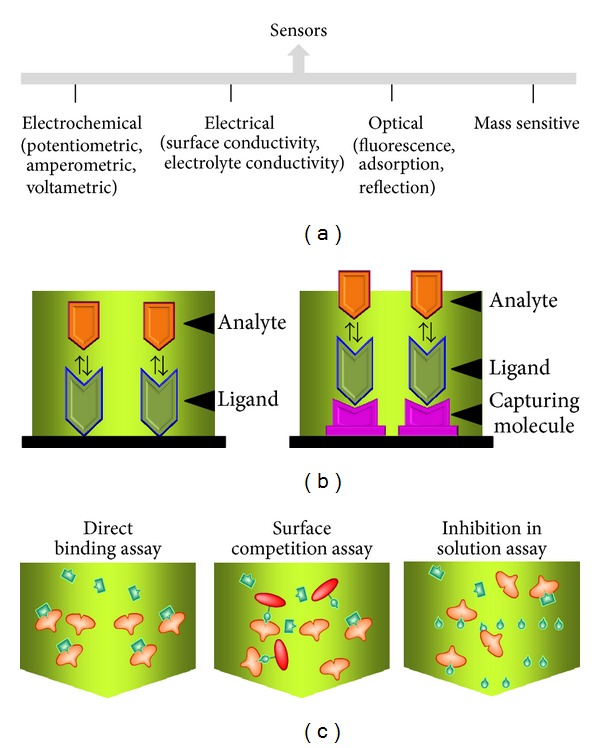
Sensing systems. (a) Types of sensors. (b) Strategy of immobilizing ligand and analyte. (c) Methods involved with ligand and analyte binding. Sensitivity depends on the interactive molecules.

A biosensor is a combination of a biological component with a physicochemical detector and it assists with analysis of biomolecular interactions. Development of an analytical system will help us to find a minute amount of biological agents within mixed compounds. Different types of sensing systems have been proposed for determining biomolecular interactions and environmental monitoring [94–98]. In general biosensors may be classified as electrochemical, electrical, optical, or mass sensitive (Figure 6(a)). The core design for sensors mainly includes three components, probe-target recognition, signal transduction, and physical readout [99]. On the sensor surfaces the lipase can be either ligand or analyte. Ligand can be immobilized directly on the sensor surface or indirectly via an immobilized surface chemical linkage (Figure 6(b)). The direct adsorption of the molecules on the sensor surface leads to rapid, simple, and cheaper strategies compared to immobilization by chemical means. Using these strategies the interactions of lipase with oil or molecules for the purpose of interactive analysis and environmental monitoring can be done by different assay formats (Figure 6(c)). Various detection and measurement methods or strategies are discussed on microbial lipases [100]. Phospholipases are potential markers for diagnosing diseases in the pancreas and coronary arteries [101, 102]. In all, free and phosphatidyl-bound choline in milk and a dietary supplement can be determined quantitatively, using a phospholipase D packed bioreactor [103]. A surface acoustic wave sensing system was generated to measure pancreatic lipase [104]. Lipase activity based on glycerol dehydrogenase/NADH oxidase was reported based on amperometric sensor [105]. Lipases can be immobilized on the sensing surface and can function as lipid biosensors for blood cholesterol determinations [106]. 

## 8. Conclusions

Fungi are capable of producing several enzymes for their survival within a wide range of substrates. Among those enzymes, lipases are predominantly used in several applications. These fat-splitting enzymes are attractive because of their applications in fields relevant to medicine and dairy industry. Lipases play a major role as the biocatalysts and microbial lipases can be produced in large scale by overexpression. The disadvantage lipase enzyme is that it continues to be active due to turnover reaction and may need to optimize the reaction condition and specificity with different sources of lipase [107]. Indeed, various methods have been proposed for the different lipases to survive under variant physical and chemical conditions. The strategies involved in the characterization of lipases were discussed here, suitable for large-scale production. The great advantage of fungal lipases is that they are easily amenable to extraction due to their extracellular nature, which will significantly reduce the cost and makes these lipases more attractive than those bacteria. Furthermore, with available sources such as LIPABASE (database for true lipases), which provides taxonomic, structural, and biochemical information, genetically engineered lipase sequences from fungal species will hasten the production, especially in the dairy industry.

## Figures and Tables

**Table 1:** Degree of utilization (%) of vegetable oils by fungal species.

Organism	Olive	Soybean	Groundnut	Cottonseed	Sunflower
*A. strictum *	20	60	40	90	90
*C. verticillata *	80	80	90	40	90
*G. candidum *	90	10	90	40	80
*M. racemosus *	50	60	60	10	50
*R. miehei *	30	90	90	80	90
*R. stolonifer *	60	70	80	20	60
*T. roseum *	90	60	80	80	80
*T. viride *	50	20	30	50	30
